# Role of NLRP3 Inflammasomes in Neurodegenerative Diseases

**DOI:** 10.5152/eurasianjmed.2023.23349

**Published:** 2023-12-01

**Authors:** Nagihan Demirtas, Busra Sahin-Mazlumoglu, Saziye Sezin Palabiyik-Yucelik

**Affiliations:** 1 Department of Pharmaceutical Toxicology, Atatürk University Faculty of Pharmacy, Erzurum, Turkey; 2 Clinical Research, Development and Design Application and Research Center, Atatürk University, Erzurum, Turkey

**Keywords:** Neurodegenerative disease, neuroinflammation, NLRP3

## Abstract

Large-scale neuronal degeneration in the human brain is a hallmark of neurodegenerative diseases. These diseases range in location and cause, but they all have neurodegenerative characteristics in common. Neurodegenerative diseases, which have almost no effective treatment options, tend to progress irreversibly and cause large socioeconomic and healthcare costs. In recent years, due to the increase in the elderly population, neurodegenerative diseases that have a risk factor with aging are becoming increasingly common. Evidence that neurodegenerative diseases, which have an important place in public health, may be caused by neuroinflammation, has led to comprehensive investigation of neurodegenerative diseases in this regard. Inflammasomes are innate immune system-associated multiproteins that regulate caspase-1 activation and induce inflammation. The NLRP3 inflammasome is the most researched inflammasome and also located in microglia, its activation mediates the maturation and secretion of the inflammatory cytokines interleukin-1beta (IL-1β) and IL-18, thus exerting its effects in the central nervous system. Within the scope of this review, experimental and human studies evaluating the role of NLRP3 inflammasome activation and the effects of its inhibition in neurodegenerative diseases frequently encountered in society have been compiled with studies from past to present.

## Introduction

The human body possesses both innate and adaptive immunity, which allow it to fend against pathogenic attacks. One of the important events that show that these immune systems are activated is inflammation.^1,2^ An intricate cellular and molecular defensive process known as inflammation is triggered by a number of different events, including stress, damage, and infection.^3^ The response of the central nervous system (CNS) to damage caused by pathogens such as infection, trauma, ischemia, and the inflammatory response that helps support the repair of nervous tissue by eliminating damaged tissues and pathogens is defined as neuroinflammation.^1,4^ During neuroinflammation, astrocytes and microglia are activated, and pro-inflammatory cytokines are released. Insufficient or excessive inflammation brought on by dysregulation in inflammatory pathways might result in chronic infections or systemic inflammatory disorders.^1^ As with many other diseases, these alterations in inflammatory processes contribute to the pathophysiology of neurodegenerative diseases. It is thought that these modifications in inflammatory mechanisms open up new research avenues for potential novel therapeutic approaches.^3^

Pathogen-associated molecular patterns (PAMPs) and damage-associated molecular patterns (DAMPs) are endogenous signals secreted due to host or environmental hazards like cellular stress and metabolic imbalance. Pattern recognition receptors (PRR) are used to mediate the recognition of these pathogenic agents.^5,6^ Activation of PRRs results in 2 ways: increase in pro-inflammatory gene expression or activation of inflammatory caspase 1.^5^ Microglia and astrocytes are the primary innate immune cells found in the CNS. Pattern recognition receptors are mostly expressed by microglia and astrocytes in the CNS, where they support innate immunity.^6-8^

As of now, retinoic acid-inducible gene-I (RIG-I)-like receptors (RLR), nucleotide-binding and oligomerization domain-like receptors (NLR), absent in melanoma 2 (AIM-2)-like receptors (ALR), and transmembrane proteins like Toll-like receptors (TLR) and C-type lectin receptors (CLR) are among the known PRR families.^9^ Pattern recognition receptors can be membrane-bound and detect signals in the extracellular environment or the endosome, as in the case of TLRs, or they can be intracellular, as in the case of NLRs and ALRs.^6,10^

Nucleotide-binding and oligomerization domain-like receptors recognize PAMPs and DAMPs and activate downstream molecules, which in turn trigger inflammation by inducing inflammasome formation.^9^ There are at least 23 identified members of the human NLR family, of which NOD-like receptor 3 (NLRP3) also recognizes metabolic stress associated with microbial particles, infection, and sterile inflammation.^5,6^ The NLRP3 inflammasome, which was first discovered in 2002, is a cytosolic multiprotein complex that triggers pro-inflammatory caspase 1, which in turn causes pyroptosis and the development and production of the inflammatory cytokines IL-18 and interleukin 1 beta (IL-1β).^2,4,6,9,11-13^ NLRP3 activation initiates the innate immune response upon PAMP and DAMP recognition.^9,14^ The cytosolic NLR, ALR, and pyrin receptors, procaspase 1, and apoptosis-associated speck-like protein (ASC), which has a caspase recruitment domain, make up the core component of an inflammasome.^6,9,13^ Composed of a caspase recruitment domain (CARD) and pyrin domain (PYD), the ASC serves as an adaptor between procaspase 1’s CARD and pyrin’s NLR. The ASC consists of a PYD and a CARD and functions as an adapter connecting NLR or the PYD of pyrin and the CARD of procaspase 1.^6,9^ The leucine-rich repeat (LRR) domain at the C-terminus, the conserved central nucleotide-binding and oligomerization domain (NACHT), and the N-terminal PYD domain make up the NLRP3 protein.^9,11^

For the NLRP3 inflammasome to function, the first signal (the priming phase) is triggered by cytokines like IL-1β and tumor necrosis factor alpha (TNF-α) or PAMPs. Among these, TLR binding to receptors activates transcription nuclear factor kappa B (NF-κB). NLRP3 activators produce PAMPs or DAMPs deliver the second signal, also referred to as the activation stage.^2,9,15^ Next, through homotypic interactions between NACHT domains, the NLRP3 protein oligomerizes. Using PYD–PYD interactions, oligomerized NLRP3 functions as a scaffold to connect with ASC and initiate the production of helical ASC filaments. Procaspase 1 engages in CARD–CARD interactions with assembled ASC.^16^ When the induced inflammasome is stimulated, PRR proteins oligomerize and preexisting procaspase-1 zymogens are drawn to the complex, which causes autoactivation and the production of active caspase 1. Following this autoactivation, pro-IL-1β and pro-IL-18 pro-peptides that are physiologically inert are transformed into mature cytokines that are released by the cell via caspase 1. Moreover, pyroptosis, a pro-inflammatory cell death that results in an early rupture of the plasma membrane, can be induced by caspase-1. Consequently, it is able to discharge its soluble intracellular portion, which sets off the inflammatory reaction.^17^

All neural cell types express the NLRP3 inflammasome under normal and pathological conditions, and NLRP3 is the most studied inflammasome in microglia in neurodegenerative diseases.^18-24^ The functions of inflammasomes in stimulating inflammation in neurodegenerative diseases are still being investigated.

## NLRP3 Inflammasome Activation and Its Relationship with Neurodegenerative Diseases

Increased loss of neurons in the CNS is a hallmark of a class of neurological conditions known as neurodegenerative diseases. Memory loss, forgetfulness, agitation, and impairment of motor functions are the main symptoms.^25^ Although the distinguishing features of these diseases include neuronal loss, reactive gliosis, and abnormal protein accumulation in disease-specific regions in the CNS, the importance of the innate and adaptive immune systems in the pathophysiology of the diseases has been emphasized in recent years.^25-27^ The CNS contains many different cell types, including neurons, endothelial cells, macrophages, and glial cells such as oligodendrocytes, astrocytes, and microglia.^27^ Microglia activation has been shown to play a role in various neurodegenerative diseases resulting in inflammatory responses.^28^ Of all the cells in the brain, microglia, which resemble resident macrophages and are found throughout the brain and spinal cord, comprise 10%-15%. In a healthy CNS, microglial cells serve as the first line of defense against pathogens.^25^ In the beginning, microglia activation protects neurons and aids in the elimination of necrotic neurons. Nevertheless, persistent overactivation by PAMPs/DAMPs, results in long-term neuroinflammatory reactions and may hasten the onset of some neurodegenerative illnesses.^25,28^ Cytokines, which lead to the formation of more inflammatory molecules by activating astrocytes and microglia, can create a feedback loop. Furthermore, the released inflammatory chemicals have the ability to stimulate other cells, such as lymphocytes and monocytes, to penetrate the blood–brain barrier and enhance CNS neuroinflammation.^3^ These alterations consequently result in neurotoxicity and accelerate the development of neurodegenerative illnesses.^25^

Microglia, the brain’s primary innate immune cells, are primarily responsible for inflammasome stimulation, but other CNS cell types have also been shown to express inflammasomes.^6,17^ The majority of NLRP3, the first inflammasome found in brain research, is found in microglia. Inflammasomes can be triggered in the CNS in response to autoimmune-mediated damage (like MS) or acute trauma (like stroke and traumatic brain injury), as well as the buildup of misfolded or aggregated proteins in the brain (like AD, ALS, and PD).^6^ While NLRP3 is the most often-researched inflammasome in the CNS, several other inflammasomes, such as NLRP1, NLRP2, NLRC4, and AIM2, are also linked to the development of neurodegenerative disorders.^29^ In general, it is believed that the creation of the chronic inflammation process, which leads to neuron loss and neurodegeneration, is greatly influenced by the increase in IL-1β and IL-18 cytokines. Thus, it is common to find elevated levels of IL-1β and IL-18 in CNS infections, brain injuries, and neurodegenerative illnesses.^30-36^ Also, these cytokines contribute to cognition, learning, and memory processes in addition to being crucial for physiological activities in the CNS.^6^ By releasing more inflammatory mediators and DAMPs, pyroptosis contributes to the pathophysiology caused by inflammasomes. It has been demonstrated that the cell death processes of apoptosis and necroptosis encourage neuroinflammation and neuronal degeneration in neurodegenerative diseases.^37^

### Multiple Sclerosis and Experimental Autoimmune Encephalomyelitis and NLRP3 Inflammasome Activation

Multiple sclerosis (MS) is a common autoimmune, demyelinating, neurodegenerative disease of the CNS. More than 2 million people worldwide have this disease, placing a serious social and economic burden on patients.^38-41^ Jean-Martin Charcot reported it for the first time in 1868.^40^ Chronic inflammatory processes in the CNS also contribute to this pathology, which occurs in 2 general phenotypes: MS (relapsing–remitting MS, RRMS), which progresses with attacks and remissions, and progressive MS.^39,42^ In MS disease, immune cell infiltration from the periphery to the blood–brain barrier causes local microglia and astrocyte activation, and the activation of these cells in the acute phase is accompanied by demyelination and plaque formation.^41,43,44^ Even though the precise origin of MS is still unknown, the disease’s main neuropathological features include inflammatory demyelination, neurodegeneration, and axonal loss. In MS patients, nerve transmission is compromised by inflammation-mediated myelinated axon loss. Cognitive and autonomic functions are also compromised, in addition to sensory and motor functions.^16,40,41^

Further in vivo or experimental research has demonstrated the involvement of NLRP3 inflammasome-related molecules in the pathophysiology of multiple sclerosis. IL-1β and IL-18 have been demonstrated to be important factors in experimental autoimmune encephalomyelitis (EAE), a commonly used mouse model of MS, and they are linked to the NLRP3 inflammasome activation pathway.^45,46^ Another factor controlling the activation of NLRP3 is the increase in extracellular ATP level. Large molecules like ATP, which are present in neurons and glia in the CNS as well as macrophages and T cells in the immune system, can pass through the nonselective plasma membrane channel known as pannexin 1. When pannexin 1 is turned on, it allows the release of ATP into the extracellular space. Thus, blocking ATP inflow via pannexin 1 prevents the activation of the NLRP3 inflammasome. Pannexin-1 channel blocking causes the start of EAE to be delayed. Furthermore, in EAE, dendritic cells’ hydrolysis of extracellular ATP prevents the activation of the NLRP3 inflammasome. In conclusion, neuroinflammatory damage in the diseased spinal cord is facilitated by a pannexin-1-mediated route (ATP release and/or NLRP3-activated inflammatory activation). Additionally, it suggests that NLRP3 activation suppression through pannexin-1 modulation can be a potential target for MS therapy.^47^ In a different study, it was shown that gasdermin d (GSDMD)-mediated inflammasome activation and pyroptosis occurred in both macrophages and microglias and myelin-forming oligodendrocytes in the CNS of MS patients and EAE. This study clearly shows that NLRP3 inflammasome activation and related pathways play a role in the pathogenesis of MS and EAE.^48^ In another study, it was shown that the MS was delayed and its severity decreased in NLRP3 (−/−) mice. It has been shown that NLRP3 plays a critical role in the induction of EAE through caspase 1-dependent cytokines.^46^ In the same study, it was shown that IL-18 was necessary for the development of EAE. It has been interpreted that the decreased IL-18 observed in NLRP3 (−/−) mice with EAE may lead to reduced EAE severity and decreased cytokines.^46^ In human studies with MS patients, it was observed that increased IL-18 levels in serum and CSF were associated with MS.^30,49^ Even, IL-1β levels in CSF correlate with cortical pathology in the very early stages of MS.^49^ In another study, miR-223-3p and NLRP3 activation, which are negative regulators of immune signaling pathways, were investigated. An increase in NLRP3 and miR-223-3p levels was detected following experimentally induced demyelination in the animal model. Then, mice were given the NLRP3 inhibitor MCC950 and endogenous miR-223-3p, and axonal damage was observed to decrease.^18^ Additionally, treatment with MCC950 not only alleviated demyelination in the brain, but also reduced oligodendrocyte loss and microglia and astrocyte activation.^50^ At the same time, studies are being carried out to develop therapeutic strategies through these pathways. The effectiveness of clemastine in the treatment of MS was evaluated on the NLRP3 pathway, and it was shown that this pathway was inhibited by clemastine treatment in the EAE model.^21^ In a study investigating the connection between NF-κB and NLRP3 in EAE, NLRP3 increased after EAE in mice. It was shown that after BAY11-7082 treatment, which is an NF-κB inhibitor, both NF-κB and NLRP3 levels decreased, thus NLRP3 playing a role in the development of EAE.^19^ In recent years, many more such compounds have been tried to reduce NLRP3 inflammasome expressions and develop new treatment approaches for MS.^22,51^ When these studies are evaluated together, it is shown that further studies are required to determine the correlation between NLRP3 and related cytokines and MS, but the importance of NLRP3 in MS physiopathology is also emphasized and it is shown that it may be an important therapeutic target in treatment.

### Alzheimer’s Disease and NLRP3 Inflammasome Activation

Alzheimer’s disease is characterized by widespread neuronal cell death, progressive memory impairment and cognitive impairments, and a common cause of dementia.^52-56^ Approximately 70% of cases of neurodegenerative illnesses are caused by AD.^56^ There is currently no recognized cause for the illness, and there is no known treatment.^57^ Its effects are irreversible. The pathophysiology of AD is commonly acknowledged to involve the buildup of aggregated Aβ to form amyloid plaques. However, the cellular events that result in the loss of neuronal function caused by plaques are not well understood, thus, it is crucial to investigate their impact on their cellular environment.^53,54,56-59^ Although its pathophysiological mechanism is not fully understood, it is increasingly accepted that neuroinflammation is one of the main mechanisms in AD.^60,61^ At the same time, several in vitro studies with microglial cells have shown that these cells secrete high amounts of cytokines upon stimulation with Aβ.^62-64^ In fact, in clinical trials conducted with nonsteroidal antiinflammatory drugs before the onset of AD, it has been suggested that inhibition of the immune response reduces the occurrence of the disease.^65^ Although microglia show repair activity by removing Aβ plaques by phagocytosis, Aβ accumulation may also trigger the activation and production of inflammatory mediators in microglial cells. In addition, the inflammatory state that begins in microglia cells may also affect other CNS cells, leading to loss of synaptic function or neuronal damage.^6^ This approach suggests that pharmaceutical intervention through immunological pathways may have considerable therapeutic promise by delaying the onset of disease through systemic suppression of inflammation or vaccination against Aβ plaques.^53^

Microglia and astrocytes can promote neuron death, pyroptosis, and the neuroinflammatory response by stimulating Aβ plaques and activating the intracellular NLRP3 inflammasome.^66^ Many recent studies have linked the development of AD to the inflammasome and inflammasome pathway-related cytokines.^53,67-70^ In a study using different experimental transgenic Alzheimer’s animal models (wild-type CD1 and APPSwe/PS1 mice), increased expression of IL-1β in microglia surrounding Aβ plaques was reported after Aβ-42 was injected into the hippocampus of the animals.^58^ In another study, peripheral blood mononuclear cells (PBMCs) isolated from blood taken from mild, moderate, and severe AD were stimulated with lipopolysaccharide (LPS) and/or Aβ, and it was shown that parameters related to the inflammatory pathway increased by PCR, confocal microscopy, and ELISA methods. It has been shown that these may be useful in the diagnosis and prognosis assessment of AD and also an indication that PBMCs may contribute to neuroinflammation in AD by crossing the blood–brain barrier.^71^ Direct inflammasome activation has not been evaluated, but it has been shown that IL-1β, one of the related cytokines, causes memory loss (amnesia) by injecting it into the dorsal hippocampus in rats, and blocking or neutralizing it with melanocortin receptors reduces cognitive impairment. Suppressing the release of these cytokines in AD, where cognitive dysfunction is observed, suggests that it will be an important target in the treatment of AD.^32^ In an in vitro study conducted with bone marrow-derived macrophages and microglia isolated from mice in which inflammasome pathway-related components were knocked out, it was shown that fibrillar Aβ, when phagocytosed by microglia, activates the NLRP3 inflammasome pathway.^53^ Also, treatment strategies were studied in AD models. A study by Ruan et al demonstrated that a curcumin-based nanoparticle effectively decreased the formation of β-amyloid plaques in APP/PS1 transgenic mice. One possible mechanism for this effect could be the inhibition of NLRP3, which in microglia tends to congregate around Aβ plaques.^72^ Cai et al conducted in vivo and in vitro studies to examine the effects of salidroside on AD. According to data obtained in in vivo studies, Aβ accumulated in the hippocampus increases IL-1β and IL-18 expression due to NLRP3 activation. It reduced pyroptosis by downregulating the expression of IL-1β and IL-18 in salidroside-treated groups*. *In vitro study results revealed also increased expression levels of NLRP3 and related proteins.^73^ Many similar studies have shown activation of NLRP3 and inflammatory cytokines due to Aβ formation.^74-77^ According to these research, NLRP3 activation is crucial for the pathogenesis and progression of AD by causing NLRP3 in microglia to trigger a chronic neuroinflammatory response. Preventive measures against NLRP3 or inflammatory cytokines resulting from its activation may reduce Aβ deposition and Tau phosphorylation due to reduced inflammatory activity in AD patients, thus ameliorating AD-associated behavioral abnormalities.

### Amyotrophic Lateral Sclerosis and NLRP3 Inflammasome Activation

The deadly neurodegenerative illness known as amyotrophic lateral sclerosis (ALS) strikes adults and is defined by a progressive loss of motor neurons in the brainstem, spinal cord, and motor cortex. This results in muscular atrophy, paralysis, and death in a matter of 1-5 years.^54,78-80^ Breathlessness, dysphagia, dysarthria, and general motor dysfunction are experienced by the patients. The chance of acquiring ALS may be influenced by environmental exposure of xenobiotics and genetic variables.^79^ Current treatments for ALS are inadequate. Therefore, there is a need to understand the mechanisms that lead to ALS pathology in order to identify new therapeutic targets that can be used to treat or prevent disease progression.^78^ Superoxide dismutase 1 (SOD1) gene is mutated in 20% of cases of familial ALS. Expression of mutant SOD1 is required in many experimental models of ALS.^81^ A mutation in SOD1 disrupts cellular homeostasis in neurons and glial cells by causing hazardous misfolding and aggregation of SOD1. Neuroinflammation mediated by glia occurs with neurodegeneration in ALS, hastening the course of the disease.^80^ While the exact cause of ALS is still unknown, new research indicates that activation of macrophages and microglia may be facilitated by the innate immune system.^78^

Neuroinflammation coexists with the loss of motor neurons in ALS patients. Meisner et al investigation revealed misfolded mutant SOD1 as an endogenous danger signal linked to disease, which microglia recognize and use to trigger inflammation through caspase-1-dependent IL-1β maturation. They showed that IL-1β maturation is correlated with the extent of mutant SOD1’s amyloid-like misfolding.^80^ Aggregates of ALS proteins are strong inducers of the immunological response in microglia.^78^ Components of the NLRP3 inflammasome have been shown to express more when humans and experimental animal models are involved.^79,82,83^ Nonetheless, conflicting findings can be found in the literature. According to a study, NLRP3 is not expressed by microglia from SOD1^G93A^ mice or human ALS patients.^83^ An additional investigation demonstrated that NLRP3 was not necessary for SOD1^G93A^-mediated caspase-1 activation and IL-1β generation in microglia.^80^ TLR4 and NF-κB were found to be elevated in the brains of SOD1^G93A^ rats that had inflammasome activation, according to Gugliandolo et al study. These are the cues that initiate the process of inflammation. It has been demonstrated that elevated levels of caspase 1, NLRP3, IL-18, and IL-1β assess brain inflammation. Finally, they showed that NLRP3 inflammasomes were activated in the brains of SOD1^G93A^ rats. This implies that inflammation is a major factor in ALS.^79^ In vivo experiments in the spinal marrow of SOD1^G93A^ mice and postmortem tissues from human sporadic ALS patients have both shown NLRP3 activation. This demonstrates that elevated amounts of mature IL-1β protein correspond with increased levels of NLRP3 inflammatory proteins in the spinal cord astrocytes of pre- and early symptomatic SOD1^G93A^ mice. According to this study, human ALS postmortem tissues have also higher levels of NLRP3, ASC, IL-18, and active caspase 1 than those of control individuals. Primary glial culture studies and double-fluorescence staining of human ALS tissue and spinal cord slices from SOD1 mice demonstrated that astrocytes are primarily responsible for NLRP3 and ASC expression.^83^ Bellezza et al’mechanism of inflammasome activation was investigated in mouse microglial cells that overexpressed hSOD1^G93A^, and it was verified in the mice’s spinal cord. For the first time, it has been demonstrated that in SOD1^G93A^ microglial cells, peroxynitrite activates caspase1 and the downstream inflammatory cascade, perhaps contributing to one of the multiple pathogenic processes associated with ALS. This study looked into the relationship between NLRP3 inflammasome activation and protein nitration brought on by an acute inflammatory stimulus (LPS) as a potential cause of chronicity in the setting of ALS. In research using SOD1^G93A^ microglial cells, LPS boosted caspase-1 activation and activated the NLRP3 inflammasome. Upon caspase-1 activation, there was a notable rise in IL-1β release. Furthermore, it has been demonstrated that LPS activates caspase 1 in SOD1^G93A^ microglial cells by causing peroxynitrite to develop. According to this study, oxidative and nitrosative stress-induced peroxynitrite production may be a key factor in the activation of inflammasomes and may be a viable target for ALS therapy.^82^ However, in another ALS model conducted with the TDP-43 mouse, enhanced regulation of microglial NLRP3 has been shown.^84^ Moreover, there was increased expression of caspase 1 and active IL-18 in the motor cortex and spinal cord of ALS patients. In TDP-43 transgenic mice, ASC and IL-18 expression increased only in microglia, while GSDMD and NLRP3 levels increased in microglia and astrocytes to a lower amount.^85^

When all of these data are taken into consideration, it appears that astrocyte NLRP3 inflammasomes are important for ALS neuroinflammation and that astrocytes and microglia secrete potentially neurotoxic cytokines to go from a neuroprotective to a pro-inflammatory phenotype.

### Parkinson’s Disease and NLRP3 Inflammasome Activation

Parkinson’s disease (PD) affects 2%-3% of those 65 and older. Globally, PD has resulted in the death of 117 000 people and has impacted the lives of 6.2 million people. Although the disease’s first symptoms include stiffness, rigidity, tremors, bradykinesia at rest, and trouble in walking, behavioral issues, depression, anxiety, and dementia are also commonly seen in the disease’s severe stages.^16,54,86-88^ According to epidemiological studies, the prevalence of Parkinson’s disease in adults over 60 in Europe and the United States is currently 1%. However, as the population ages and life expectancy rises, the number of Parkinson’s patients is predicted to rise by more than 50% by 2030. There is currently no theory to explain the cause of dopaminergic neuron loss and no apparent mechanism linking to the pathophysiology of PD. Still, a growing body of research indicates that multiple pathogenic factors contribute to PD.^86^ Among these, one of the key pathogenic causes is increased alpha-synuclein (α-Syn) aggregation.^59,86^ This disease results in the formation of Lewy bodies, which are fibrillary misfolded α-Syn aggregates that frequently cause the death of dopaminergic neurons.^89^ Damaged neurons produce aggregated α-Syn into the extracellular area, which then activates microglial cells.^90^ It is believed that the development of PD is significantly influenced by inflammatory responses mediated by inflammatory cytokines, such as IL-1β generated from microglial cells.^87^

alpha-Syn aggregates can induce NLRP3 inflammasome activation by activating microglial cells, which exacerbates neuroinflammation in PD.^87,91^ Yan et al found in 2015 that NLRP3^−/−^ animals were more resistant to developed PD in a mouse model produced by loss of nigral dopaminergic neurons triggered by treatment with 1-methyl-4-phenyl-1,2,3,6-tetrahydropyridine. This provides in vivo proof of the connection between PD and the NLRP3 inflammasome.^92^ Similar results were obtained from another study, which demonstrated that NLRP3^−/−^ mice were more resistant to the development of PD in mice that had rotenone-induced parkinsonism.^93^ For the first time, it was demonstrated in the 2016 study by Zhou et al. that NLRP3 inflammasome activation plays a crucial role in the pathophysiology of PD and induces microglia-mediated neuroinflammation and dopaminergic neuronal degeneration. Nevertheless, it has also been demonstrated that alpha-Syn activates the NLRP3 inflammasome by lysosome damage and microglial endocytosis. Moreover, data from both in vitro and in vivo experiments have demonstrated that miR-7, a microRNA known to play a role in PD via controlling α-Syn, targets NLRP3 expression and prevents the activation of the NLRP3 inflammasome in PD model mice.^94^

The 2 most common animal models of PD are those produced by 6-hydroxydopamine (6-OHDA) and LPS. Many clinical and physiological characteristics, including a decrease in dopamine activity and its content in the brain, are similar to human PD. These models include degeneration and death of dopamine neurons as well as proliferation and activation of glial cells. It has been demonstrated that dopaminergic neurons can be preserved in PD models produced by 6-OHDA and LPS by blocking the NLRP3 cascade axis activity through the administration of a Ac-YVAD-CMK, caspase-1 inhibitor.^95^ In patients with autosomal recessive early-onset PD, mutations were detected in genes associated with the disease. In the studies of Mouton-Liger et al in 2018, it was shown that patients with PARK2 mutations exhibited an aggravated NLRP3 inflammasome response. It has been demonstrated that the sulfonylurea-containing substance MCC950 corrects dopaminergic degeneration by preventing NLRP3’s canonical and noncanonical activation as well as IL-1β activation.^96^ Apart from these studies conducted in experimental animal models, histological studies in human studies conducted on PD have also found that NLRP3 expression is increased in the mesencephalic neurons of PD patients.^97^ According to a different study, serum IL-1β and caspase-1 activity and IL-1β and IL-18 levels in the cerebrospinal fluid increased in Parkinson’s patients.^94,98^ In another in vivo human study, the NLRP3 inflammasome and ASC and caspase 1 are increased in microglia in areas of dopaminergic cell loss in PD patients.^99^

TLR4, one of the main innate immune receptors activated in PD, and its downstream molecule, TAK1, can stimulate neuroinflammatory responses. In Quan et al’s study, it was shown that the protein expression of NLRP3, active caspase 1, GSDMD-N, and mature IL-1β was significantly increased in the PD rat model. It has also been shown for the first time that interferon regulatory factor 7 (IRF7) increases in PD and the regulatory relationship between NLRP3.^23^ In in vivo, in vitro, and human studies, Li et al tried to elucidate the molecular mechanism between nuclear receptor-related 1 (NURR1) and miR-30e-5p and NLRP3 in microglia. Microglia express the ligand-activated transcription factor NURR1, which may be a PD susceptibility gene. Nuclear receptor-related-1 expression has anti-inflammatory properties, since it has a negative correlation with the expression levels of inflammatory cytokines. NLRP3 and IL-1β levels were found to be high in blood samples collected from patients with PD. The absence of NURR1 led to increased alpha-syn aggregation and NLRP3 activation in the mouse model.^100^ Studies were also conducted for the treatment of PD through this pathway. It was shown that the neurotoxic effect of levodopa, which is used for treatment of PD due to excessive accumulation, was reduced when given together with *Nardostachys jatamansi *(NJ, Chinese medicine). It has been shown that inhibition of NLRP3 activation by IL-1β and IL-18 and caspase-1 inhibition underlies this effect.^20^

Therefore, it may be concluded that the NLRP3 inflammasome is essential to both the pathophysiology and inflammation-induced degeneration of dopaminergic neurons in PD. In order to alleviate neuroinflammation in PD, the role of these pathways in the pathogenesis of the disease should be fully understood and revealed. Upon confirmation, inhibitors of the NLRP3 inflammasome pathway could potentially serve as a significant therapeutic target for the creation of novel treatment models aimed at treating the disease during its initial phases. And these molecules may help lessen the symptoms of Parkinson’s disease and demonstrate some neuroprotection.

## Conclusion

Inflammasomes are currently thought to be key regulators of innate immunity and play a crucial role in the host’s defense against infections and stressful environments. On the other hand, the activation of these multiprotein complexes also adds to the detrimental environment that fosters the emergence of neurodegenerative illnesses. Neurodegenerative diseases are one of the main causes of death worldwide, affected by many factors such as age, genetics, and trauma. Although it negatively affects the quality of human life, treatment options are unfortunately few and generally aimed at improving the quality of life or slowing down the progression of the disease. Although it has been thought for years that these diseases are caused by the accumulation of various proteins in the brain, studies have shown that the innate and adaptive immune system may also be related to this with microglial cells. Within the scope of this review, we examined and compiled the role of microglial NLRP3 inflammasome activation and related inflammation parameters in neurodegenerative diseases with a high incidence in society. Many studies focusing on understanding the role of inflammasomes in neurodegenerative diseases have taken their place in the literature. And looking at the evidence provided by these studies, there is a link between NLRP3 and neurodegenerative diseases, but its exact role and mechanisms need to be proven in further studies. Considering these studies, it becomes clear that inhibiting neuroinflammation, which is thought to cause these neurodegenerative diseases, may be a therapeutic target. Although the role of inflammasomes, especially NLRP3, has been extensively investigated, there is a gap in the literature regarding their effectiveness and how to use them in the treatment of these diseases. These studies are important to provide more evidence and targets for understanding the mechanisms of diseases and researching new treatment methods. Activation of the NLRP3 inflammasome and related parameters in the development of neurodegenerative diseases should be investigated in detail, and after the roles of all these parameters in the development of these diseases are precisely defined, they should be transformed into a therapeutic strategy in studies on prevention and treatment.

## References

[1] Pirzada RH Javaid N Choi S . The roles of the NLRP3 inflammasome in neurodegenerative and metabolic diseases and in relevant advanced therapeutic interventions. Genes. 2020;11(2):131. (10.3390/genes11020131) 32012695 PMC7074480

[2] Aydin P Magden ZBA Uzuncakmak SK , et al. Avanafil as a novel therapeutic agent against LPS-induced acute lung injury via increasing cGMP to downregulate the TLR4-NF-kappaB-NLRP3 inflammasome signaling pathway. Lung. 2022;200(5):561 572. (10.1007/s00408-022-00564-9) 36040529

[3] Liu L Chan C . The role of inflammasome in Alzheimer’s disease. Ageing Res Rev. 2014;15:6 15. (10.1016/j.arr.2013.12.007) 24561250 PMC4029867

[4] Leng F Edison P . Neuroinflammation and microglial activation in Alzheimer disease: where do we go from here? Nat Rev Neurol. 2021;17(3):157 172. (10.1038/s41582-020-00435-y) 33318676

[5] Aksoy B . Derinin doğal bağışıklık sistemi. TURKDERM. 2013;47(1):2 11. (10.4274/turkderm.47.s1)

[6] Voet S Srinivasan S Lamkanfi M van Loo G . Inflammasomes in neuroinflammatory and neurodegenerative diseases. EMBO Mol Med. 2019;11(6):e10248. (10.15252/emmm.201810248) 31015277 PMC6554670

[7] Stephenson J Nutma E van der Valk P Amor S . Inflammation in CNS neurodegenerative diseases. Immunology. 2018;154(2):204 219. (10.1111/imm.12922) 29513402 PMC5980185

[8] Atmaca HT Kul O Karakuş E Terzi OS Canpolat S Anteplioğlu T . Astrocytes, microglia/macrophages, and neurons expressing toll-like receptor 11 contribute to innate immunity against encephalitic Toxoplasma gondii infection. Neuroscience. 2014;269:184 191. (10.1016/j.neuroscience.2014.03.049) 24704432

[9] Ispir FB Yucelik-Palabiyik SS . The role of Inflammasomes in liver toxicity. Hacettepe Univ J Fac Pharm. 2019;39(2):90 97

[10] Takeuchi O Akira S . Pattern recognition receptors and inflammation. Cell. 2010;140(6):805 820. (10.1016/j.cell.2010.01.022) 20303872

[11] Barczuk J Siwecka N Lusa W Rozpędek-Kamińska W Kucharska E Majsterek I . Targeting NLRP3-mediated neuroinflammation in Alzheimer’s disease treatment. Int J Mol Sci. 2022;23(16) (10.3390/ijms23168979) PMC9409081 36012243

[12] Aydin P Aksakalli-Magden ZB Civelek MS , et al. The melatonin agonist ramelteon attenuates bleomycin-induced lung fibrosis by suppressing the NLRP3/TGF-Beta1/HMGB1 signaling pathway. Adv Med Sci. 2023;68(2):322 331. (10.1016/j.advms.2023.09.004) 37716182

[13] Bilen A Calik I Yayla M , et al. Does daily fasting shielding kidney on hyperglycemia-related inflammatory cytokine via TNF-alpha, NLRP3, TGF-beta1 and VCAM-1 mRNA expression. Int J Biol Macromol. 2021;190:911 918. (10.1016/j.ijbiomac.2021.08.216) 34492249

[14] Yoladi FB Yucelik SS Baydar T Halici Z . P26-09: Protective effects of coenzyme Q10 and idebenone on ethanol induced liver fibrosis through inhibiting NLRP3/Caspase-1/IL-1β inflammasome pathway. Toxicol Lett. 2023;384:S295 S296. (10.1016/S0378-4274(23)00959-1)

[15] Şimşek H Akaras N Gür C Küçükler S Kandemir FM . Beneficial effects of chrysin on Cadmium-induced nephrotoxicity in rats: modulating the levels of Nrf2/HO-1, RAGE/NLRP3, and caspase-3/Bax/Bcl-2 signaling pathways. Gene. 2023;875:147502. (10.1016/j.gene.2023.147502) 37224935

[16] Lahooti B Chhibber T Bagchi S Varahachalam SP Jayant RD . Therapeutic role of inflammasome inhibitors in neurodegenerative disorders. Brain Behav Immun. 2021;91:771 783. (10.1016/j.bbi.2020.11.004) 33157255

[17] Yardim A Gur C Comakli S , et al. Investigation of the effects of berberine on bortezomib-induced sciatic nerve and spinal cord damage in rats through pathways involved in oxidative stress and neuro-inflammation. Neurotoxicology. 2022;89:127 139. (10.1016/j.neuro.2022.01.011) 35121005

[18] Galloway DA Carew SJ Blandford SN , et al. Investigating the NLRP3 inflammasome and its regulator miR-223-3p in multiple sclerosis and experimental demyelination. J Neurochem. 2022;163(2):94 112. (10.1111/jnc.15650) 35633501

[19] Lang Y Chu F Liu L , et al. Potential role of BAY11-7082, a NF-κB blocker inhibiting experimental autoimmune encephalomyelitis in C57BL/6J mice via declining NLRP3 inflammasomes. Clin Exp Immunol. 2022;207(3):378 386. (10.1093/cei/uxab022) 35553640 PMC9113142

[20] Li J Yu J Guo J , et al. Nardostachys jatamansi and levodopa combination alleviates Parkinson’s disease symptoms in rats through activation of Nrf2 and inhibition of NLRP3 signaling pathways. Pharm Biol. 2023;61(1):1175 1185. (10.1080/13880209.2023.2244176) 37559448 PMC10416743

[21] Motawi TK El-Maraghy SA Kamel AS Said SE Kortam MA . Modulation of p38 MAPK and Nrf2/HO-1/NLRP3 inflammasome signaling and pyroptosis outline the anti-neuroinflammatory and remyelinating characters of clemastine in EAE rat model. Biochem Pharmacol. 2023;209:115435. (10.1016/j.bcp.2023.115435) 36720356

[22] Naeem AG El-Naga RN Michel HE . Nebivolol elicits a neuroprotective effect in the cuprizone model of multiple sclerosis in mice: emphasis on M1/M2 polarization and inhibition of NLRP3 inflammasome activation. Inflammopharmacology. 2022;30(6):2197 2209. (10.1007/s10787-022-01045-4) 35948811 PMC9700639

[23] Quan W Liu Y Li J , et al. Investigating the TLR4/TAK1/IRF7 axis in NLRP3-Mediated pyroptosis in Parkinson’s disease. Inflammation. 2023:1 17. (10.1007/s10753-023-01918-y) 37930487

[24] Keskin H Keskin F Tavaci T , et al. Neuroprotective effect of Roflumilast under cerebral ischaemia/reperfusion injury in juvenile rats through NLRP-mediated inflammatory response inhibition. Clin Exp Pharmacol Physiol. 2021;48(8):1103 1110. (10.1111/1440-1681.13493) 33686709

[25] Wu AG Zhou XG Qiao G , et al. Targeting microglial autophagic degradation in NLRP3 inflammasome-mediated neurodegenerative diseases. Ageing Res Rev. 2021;65:101202. (10.1016/j.arr.2020.101202) 33161129

[26] Pascoal TA Benedet AL Ashton NJ , et al. Microglial activation and tau propagate jointly across Braak stages. Nat Med. 2021;27(9):1592 1599. (10.1038/s41591-021-01456-w) 34446931

[27] Mason HD McGavern DB . How the immune system shapes neurodegenerative diseases. Trends Neurosci. 2022;45(10):733 748. (10.1016/j.tins.2022.08.001) 36075783 PMC9746609

[28] Xu L He D Bai Y . Microglia-mediated inflammation and neurodegenerative disease. Mol Neurobiol. 2016;53(10):6709 6715. (10.1007/s12035-015-9593-4) 26659872

[29] Heneka MT McManus RM Latz E . Inflammasome signalling in brain function and neurodegenerative disease. Nat Rev Neurosci. 2018;19(10):610 621. (10.1038/s41583-018-0055-7) 30206330

[30] Chen YC Chen SD Miao L , et al. Serum levels of interleukin (IL)-18, IL-23 and IL-17 in Chinese patients with multiple sclerosis. J Neuroimmunol. 2012;243(1-2):56 60. (10.1016/j.jneuroim.2011.12.008) 22230485

[31] Craft JM Watterson DM Hirsch E Van Eldik LJ . Interleukin 1 receptor antagonist knockout mice show enhanced microglial activation and neuronal damage induced by intracerebroventricular infusion of human β-amyloid. J Neuroinflammation. 2005;2(1):15. (10.1186/1742-2094-2-15) 15967035 PMC1190207

[32] Gonzalez PV Schiöth HB Lasaga M Scimonelli TN . Memory impairment induced by IL-1beta is reversed by alpha-MSH through central melanocortin-4 receptors. Brain Behav Immun. 2009;23(6):817 822. (10.1016/j.bbi.2009.03.001) 19275930

[33] Avci S Gunaydin S Ari NS , et al. Cerebrolysin alleviating effect on glutamate-mediated neuroinflammation via glutamate transporters and oxidative stress. J Mol Neurosci. 2022;72(11):2292 2302. (10.1007/s12031-022-02078-8) 36333611

[34] Yıldız MO Çelik H Caglayan C , et al. Neuromodulatory effects of hesperidin against sodium fluoride-induced neurotoxicity in rats: involvement of neuroinflammation, endoplasmic reticulum stress, apoptosis and autophagy. Neurotoxicology. 2022;90:197 204. (10.1016/j.neuro.2022.04.002) 35413380

[35] Cakir M Zengin S Calikoglu C , et al. Protective role of interferon-Β in experimentally induced Perıpheral nerve injury in rats. Acta Med Mediterr. 2015;31:973

[36] Mert H Kerem Ö Mıs L Yıldırım S Mert N . Effects of protocatechuic acid against cisplatin-induced neurotoxicity in rat brains: an experimental study. Int J Neurosci. 2022:1 10. (10.1080/00207454.2022.2147430) 36525373

[37] Zhang S Tang MB Luo HY Shi CH Xu YM . Necroptosis in neurodegenerative diseases: a potential therapeutic target. Cell Death Dis. 2017;8(6):e2905. (10.1038/cddis.2017.286) 28661482 PMC5520937

[38] Cui Y Yu H Bu Z Wen L Yan L Feng J . Focus on the role of the NLRP3 inflammasome in multiple sclerosis: pathogenesis, diagnosis, and therapeutics. Front Mol Neurosci. 2022;15:894298. (10.3389/fnmol.2022.894298) 35694441 PMC9175009

[39] Şİmşek F ÖztÜrk N . Dclk-1 level in multiple sclerosis patients and its correlation with clinic. Mult Scler Relat Disord. 2020;43:102179. (10.1016/j.msard.2020.102179) 32470859

[40] Balkan E Bilge N . Expression levels of IL-17/IL-23 cytokine-targeting microRNAs 20, 21, 26, 155, and Let-7 in patients with relapsing-remitting multiple sclerosis. Neurol Res. 2021;43(9):778 783. (10.1080/01616412.2021.1935099) 34130607

[41] Bilge N Simsek F Yevgi R Ceylan M Askın S . Low serum alpha-synuclein and oligomer alpha-synuclein levels in multiple sclerosis patients. J Neuroimmunol. 2020;350:577432. (10.1016/j.jneuroim.2020.577432) 33220655

[42] Barclay W Shinohara ML . Inflammasome activation in multiple sclerosis and experimental autoimmune encephalomyelitis (EAE). Brain Pathol. 2017;27(2):213 219. (10.1111/bpa.12477) 27997058 PMC8029098

[43] Baecher-Allan C Kaskow BJ Weiner HL . Multiple sclerosis: mechanisms and immunotherapy. Neuron. 2018;97(4):742 768. (10.1016/j.neuron.2018.01.021) 29470968

[44] Kuhlmann T Moccia M Coetzee T , et al. Multiple sclerosis progression: time for a new mechanism-driven framework. Lancet Neurol. 2023;22(1):78 88. (10.1016/S1474-4422(22)00289-7) 36410373 PMC10463558

[45] Inoue M Williams KL Gunn MD Shinohara ML . NLRP3 inflammasome induces chemotactic immune cell migration to the CNS in experimental autoimmune encephalomyelitis. Proc Natl Acad Sci U S A. 2012;109(26):10480 10485. (10.1073/pnas.1201836109) 22699511 PMC3387125

[46] Gris D Ye Z Iocca HA , et al. NLRP3 plays a critical role in the development of experimental autoimmune encephalomyelitis by mediating Th1 and Th17 responses. J Immunol. 2010;185(2):974 981. (10.4049/jimmunol.0904145) 20574004 PMC3593010

[47] Lutz SE González-Fernández E Ventura JC , et al. Contribution of pannexin1 to experimental autoimmune encephalomyelitis. PLoS One. 2013;8(6):e66657. (10.1371/journal.pone.0066657) 23885286 PMC3688586

[48] McKenzie BA Mamik MK Saito LB , et al. Caspase-1 inhibition prevents glial inflammasome activation and pyroptosis in models of multiple sclerosis. Proc Natl Acad Sci U S A. 2018;115(26):E6065 E6074. (10.1073/pnas.1722041115) 29895691 PMC6042136

[49] Seppi D Puthenparampil M Federle L , et al. Cerebrospinal fluid IL-1beta correlates with cortical pathology load in multiple sclerosis at clinical onset. J Neuroimmunol. 2014;270(1-2):56 60. (10.1016/j.jneuroim.2014.02.014) 24657029

[50] Hou B Yin J Liu S , et al. Inhibiting the NLRP3 inflammasome with MCC950 alleviates neurological impairment in the brain of EAE mice. Mol Neurobiol. 2023:1 13. (10.1007/s12035-023-03618-y) PMC10896958 37702910

[51] Chen C Zhou Y Ning X , et al. Directly targeting ASC by lonidamine alleviates inflammasome-driven diseases. J Neuroinflammation. 2022;19(1):315. (10.1186/s12974-022-02682-w) 36577999 PMC9798610

[52] Khan S Barve KH Kumar MS . Recent advancements in pathogenesis, diagnostics and treatment of Alzheimer’s disease. Curr Neuropharmacol. 2020;18(11):1106 1125. (10.2174/1570159X18666200528142429) 32484110 PMC7709159

[53] Halle A Hornung V Petzold GC , et al. The NALP3 inflammasome is involved in the innate immune response to amyloid-beta. Nat Immunol. 2008;9(8):857 865. (10.1038/ni.1636) 18604209 PMC3101478

[54] Danisman B Kelek SE Aslan M . Resveratrol in neurodegeneration, in neurodegenerative diseases, and in the redox biology of the mitochondria. Psychiatry Clin Psychopharmacol. 2023;33(2):147 155. (10.5152/pcp.2023.23633) 38765928 PMC11082578

[55] Ertekin A Demir R Ozdemir G Özel L Özyıldırım E Ulvi H . An investigation of the risk factors and prevalence of Alzheimer’s disease in the eastern region of turkey: a population based door-to-door survey. Eur J Gen Med. 2015;12(2):144 151

[56] Turkez H Arslan ME Barboza JN Kahraman CY de Sousa DP Mardinoğlu A . Therapeutic potential of ferulic acid in Alzheimer’s disease. Curr Drug Deliv. 2022;19(8):860 873. (10.2174/1567201819666211228153801) 34963433

[57] Pirim I Erdogan AR Tan U . N-terminal heterogenicity of amyloid protein examined in Alzheimer’s disease. Int J Neurosci. 2004;114(1):75 81. (10.1080/00207450490249455) 14660069

[58] Simard AR Soulet D Gowing G Julien JP Rivest S . Bone marrow-derived microglia play a critical role in restricting senile plaque formation in Alzheimer’s disease. Neuron. 2006;49(4):489 502. (10.1016/j.neuron.2006.01.022) 16476660

[59] Lam S Bayraktar A Zhang C , et al. A systems biology approach for studying neurodegenerative diseases. Drug Discov Today. 2020;25(7):1146 1159. (10.1016/j.drudis.2020.05.010) 32442631

[60] Akiyama H Barger S Barnum S , et al. Inflammation and Alzheimer’s disease. Neurobiol Aging. 2000;21(3):383 421. (10.1016/s0197-4580(00)00124-x) 10858586 PMC3887148

[61] Sala Frigerio C De Strooper B . Alzheimer’s disease mechanisms and emerging roads to novel therapeutics. Annu Rev Neurosci. 2016;39:57 79. (10.1146/annurev-neuro-070815-014015) 27050320

[62] Chen JM Li QW Jiang GX Liu JS Cheng Q . IL-18 induced IL-23/IL-17 expression impairs Aβ clearance in cultured THP-1 and BV2 cells. Cytokine. 2019;119:113 118. (10.1016/j.cyto.2019.03.003) 30903865

[63] Liu Y Dai Y Li Q , et al. Beta-amyloid activates NLRP3 inflammasome via TLR4 in mouse microglia. Neurosci Lett. 2020;736:135279. (10.1016/j.neulet.2020.135279) 32726591

[64] Nopparat C Boontor A Kutpruek S Govitrapong P . The role of melatonin in amyloid beta-induced inflammation mediated by inflammasome signaling in neuronal cell lines. Sci Rep. 2023;13(1):17841. (10.1038/s41598-023-45220-1) 37857668 PMC10587142

[65] Yip AG Green RC Huyck M Cupples LA Farrer LA , MIRAGE Study Group. Nonsteroidal anti-inflammatory drug use and Alzheimer’s disease risk: the Mirage Study. BMC Geriatr. 2005;5:2. (10.1186/1471-2318-5-2) 15647106 PMC546007

[66] Liang T Zhang Y Wu S Chen Q Wang L . The role of NLRP3 inflammasome in Alzheimer’s disease and potential therapeutic targets. Front Pharmacol. 2022;13:845185. (10.3389/fphar.2022.845185) 35250595 PMC8889079

[67] Flores J Noël A Fillion ML LeBlanc AC . Therapeutic potential of Nlrp1 inflammasome, caspase-1, or caspase-6 against Alzheimer disease cognitive impairment. Cell Death Differ. 2022;29(3):657 669. (10.1038/s41418-021-00881-1) 34625662 PMC8901623

[68] Heneka MT Kummer MP Stutz A , et al. NLRP3 is activated in Alzheimer’s disease and contributes to pathology in APP/PS1 mice. Nature. 2013;493(7434):674 678. (10.1038/nature11729) 23254930 PMC3812809

[69] Zhang M Wu Y Gao R Chen X Chen R Chen Z . Glucagon-like peptide-1 analogs mitigate neuroinflammation in Alzheimer’s disease by suppressing NLRP2 activation in astrocytes. Mol Cell Endocrinol. 2022;542:111529. (10.1016/j.mce.2021.111529) 34906628

[70] Wu PJ Hung YF Liu HY Hsueh YP . Deletion of the inflammasome sensor Aim2 mitigates Aβ deposition and microglial activation but increases inflammatory cytokine expression in an Alzheimer disease mouse model. Neuroimmunomodulation. 2017;24(1):29 39. (10.1159/000477092) 28618410

[71] Saresella M La Rosa F Piancone F , et al. The NLRP3 and NLRP1 inflammasomes are activated in Alzheimer’s disease. Mol Neurodegener. 2016;11:23. (10.1186/s13024-016-0088-1) 26939933 PMC4778358

[72] Ruan Y Xiong Y Fang W , et al. Highly sensitive curcumin-conjugated nanotheranostic platform for detecting amyloid-beta plaques by magnetic resonance imaging and reversing cognitive deficits of Alzheimer’s disease via NLRP3-inhibition. J Nanobiotechnology. 2022;20(1):322. (10.1186/s12951-022-01524-4) 35836190 PMC9281113

[73] Cai Y Chai Y Fu Y , et al. Salidroside ameliorates Alzheimer’s disease by targeting NLRP3 inflammasome-mediated pyroptosis. Front Aging Neurosci. 2021;13:809433. (10.3389/fnagi.2021.809433) 35126093 PMC8814655

[74] Jung ES Suh K Han J , et al. Amyloid-β activates NLRP3 inflammasomes by affecting microglial immunometabolism through the Syk-AMPK pathway. Aging Cell. 2022;21(5):e13623. (10.1111/acel.13623) 35474599 PMC9124305

[75] Kumar S Budhathoki S Oliveira CB , et al. Role of the caspase-8/RIPK3 axis in Alzheimer’s disease pathogenesis and Aβ-induced NLRP3 inflammasome activation. JCI Insight. 2023;8(3). (10.1172/jci.insight.157433) PMC9977425 36602874

[76] Yang T Zhang L Shang Y , et al. Concurrent suppression of Aβ aggregation and NLRP3 inflammasome activation for treating Alzheimer’s disease. Chem Sci. 2022;13(10):2971 2980. (10.1039/d1sc06071f) 35382471 PMC8905858

[77] Lin W Li Z Liang G , et al. TNEA therapy promotes the autophagic degradation of NLRP3 inflammasome in a transgenic mouse model of Alzheimer’s disease via TFEB/TFE3 activation. J Neuroinflammation. 2023;20(1):21. (10.1186/s12974-023-02698-w) 36732771 PMC9896717

[78] Deora V Lee JD Albornoz EA , et al. The microglial NLRP3 inflammasome is activated by amyotrophic lateral sclerosis proteins. Glia. 2020;68(2):407 421. (10.1002/glia.23728) 31596526

[79] Gugliandolo A Giacoppo S Bramanti P Mazzon E . NLRP3 inflammasome activation in a transgenic amyotrophic lateral sclerosis model. Inflammation. 2018;41(1):93 103. (10.1007/s10753-017-0667-5) 28936769

[80] Meissner F Molawi K Zychlinsky A . Mutant superoxide dismutase 1-induced IL-1beta accelerates ALS pathogenesis. Proc Natl Acad Sci U S A. 2010;107(29):13046 13050. (10.1073/pnas.1002396107) 20616033 PMC2919927

[81] Kaur SJ McKeown SR Rashid S . Mutant SOD1 mediated pathogenesis of amyotrophic lateral sclerosis. Gene. 2016;577(2):109 118. (10.1016/j.gene.2015.11.049) 26657039

[82] Bellezza I Grottelli S Costanzi E , et al. Peroxynitrite activates the NLRP3 inflammasome cascade in SOD1(G93A) mouse model of amyotrophic lateral sclerosis. Mol Neurobiol. 2018;55(3):2350 2361. (10.1007/s12035-017-0502-x) 28357805

[83] Johann S Heitzer M Kanagaratnam M , et al. NLRP3 inflammasome is expressed by astrocytes in the SOD1 mouse model of ALS and in human sporadic ALS patients. Glia. 2015;63(12):2260 2273. (10.1002/glia.22891) 26200799

[84] Ince PG Highley JR Kirby J , et al. Molecular pathology and genetic advances in amyotrophic lateral sclerosis: an emerging molecular pathway and the significance of glial pathology. Acta Neuropathol. 2011;122(6):657 671. (10.1007/s00401-011-0913-0) 22105541

[85] Van Schoor E Ospitalieri S Moonen S , et al. Increased pyroptosis activation in white matter microglia is associated with neuronal loss in ALS motor cortex. Acta Neuropathol. 2022;144(3):393 411. (10.1007/s00401-022-02466-9) 35867112

[86] Huang Q Yang P Liu Y Ding J Lu M Hu G . The interplay between alpha-synuclein and NLRP3 inflammasome in Parkinson’s disease. Biomed Pharmacother. 2023;168:115735. (10.1016/j.biopha.2023.115735) 37852103

[87] Wang S Yuan YH Chen NH Wang HB . The mechanisms of NLRP3 inflammasome/pyroptosis activation and their role in Parkinson’s disease. Int Immunopharmacol. 2019;67:458 464. (10.1016/j.intimp.2018.12.019) 30594776

[88] Altinoz MA Elmaci İ Hacimuftuoglu A Ozpinar A Hacker E Ozpinar A . PPARdelta and its ligand erucic acid may act anti-tumoral, neuroprotective, and myelin protective in neuroblastoma, glioblastoma, and Parkinson’s disease. Mol Aspects Med. 2021;78:100871. (10.1016/j.mam.2020.100871) 32703610

[89] Shulman JM De Jager PL Feany MB . Parkinson’s disease: genetics and pathogenesis. Annu Rev Pathol. 2011;6:193 222. (10.1146/annurev-pathol-011110-130242) 21034221

[90] Croisier E Moran LB Dexter DT Pearce RK Graeber MB . Microglial inflammation in the parkinsonian substantia nigra: relationship to alpha-synuclein deposition. J Neuroinflammation. 2005;2(1):14. (10.1186/1742-2094-2-14) 15935098 PMC1177985

[91] Gustot A Gallea JI Sarroukh R Celej MS Ruysschaert JM Raussens V . Amyloid fibrils are the molecular trigger of inflammation in Parkinson’s disease. Biochem J. 2015;471(3):323 333. (10.1042/BJ20150617) 26272943

[92] Yan Y Jiang W Liu L , et al. Dopamine controls systemic inflammation through inhibition of NLRP3 inflammasome. Cell. 2015;160(1-2):62 73. (10.1016/j.cell.2014.11.047) 25594175

[93] Martinez EM Young AL Patankar YR , et al. Editor’s highlight: Nlrp3 is required for inflammatory changes and nigral cell loss resulting from chronic intragastric rotenone exposure in mice. Toxicol Sci. 2017;159(1):64 75. (10.1093/toxsci/kfx117) 28903492 PMC5837210

[94] Zhou Y Lu M Du RH , et al. MicroRNA-7 targets Nod-like receptor protein 3 inflammasome to modulate neuroinflammation in the pathogenesis of Parkinson’s disease. Mol Neurodegener. 2016;11(1):28. (10.1186/s13024-016-0094-3) 27084336 PMC4833896

[95] Mao ZJ Liu CC Ji SQ , et al. The NLRP3 inflammasome is involved in the pathogenesis of Parkinson’s disease in rats. Neurochem Res. 2017;42(4):1104 1115. (10.1007/s11064-017-2185-0) 28247334

[96] Mouton-Liger F Rosazza T Sepulveda-Diaz J , et al. P arkin deficiency modulates NLRP 3 inflammasome activation by attenuating an A 20-dependent negative feedback loop. Glia. 2018;66(8):1736 1751. (10.1002/glia.23337) 29665074 PMC6190839

[97] von Herrmann KM Salas LA Martinez EM , et al. NLRP3 expression in mesencephalic neurons and characterization of a rare NLRP3 polymorphism associated with decreased risk of Parkinson’s disease. npj Parkinsons Dis. 2018;4:24. (10.1038/s41531-018-0061-5) 30131971 PMC6093937

[98] Zhang P Shao XY Qi GJ , et al. Cdk5-dependent activation of neuronal Inflammasomes in Parkinson’s disease. Mov Disord. 2016;31(3):366 376. (10.1002/mds.26488) 26853432

[99] Gordon R Albornoz EA Christie DC , et al. Inflammasome inhibition prevents alpha-synuclein pathology and dopaminergic neurodegeneration in mice. Sci Transl Med. 2018;10(465). (10.1126/scitranslmed.aah4066) PMC6483075 30381407

[100] Li T Tan X Tian L , et al. The role of Nurr1-miR-30e-5p-NLRP3 axis in inflammation-mediated neurodegeneration: insights from mouse models and patients’ studies in Parkinson’s disease. J Neuroinflammation. 2023;20(1):274. (10.1186/s12974-023-02956-x) 37990334 PMC10664369

